# Peeling the Layers of Caddisfly Diversity on a Longitudinal Gradient in Karst Freshwater Habitats Reveals Community Dynamics and Stability

**DOI:** 10.3390/insects12030234

**Published:** 2021-03-10

**Authors:** Ivana Pozojević, Marija Ivković, Katarina Ana Cetinić, Ana Previšić

**Affiliations:** 1 Department of Biology, Division of Zoology, Faculty of Science, University of Zagreb, Rooseveltov trg 6, 10000 Zagreb, Croatia; marija.ivkovic@biol.pmf.hr; 2 Ruđer Bošković Institute, Bijenička cesta 54, 10000 Zagreb, Croatia; katarina.cetinic@irb.hr

**Keywords:** diversity profile, non-naïve diversity profile, similarity matrix, sensitivity parameter *q*, climate change vulnerability

## Abstract

**Simple Summary:**

Freshwater biodiversity is facing a severe crisis due to many different human-caused impacts, such as climate change, pollution, habitat alterations, etc. Aquatic insects are one of the most important bioindicators used in freshwater ecological quality assessment systems, yet knowledge on diversity dynamics of their communities is incomplete. In the current study, we compare and evaluate performance of different diversity measures, i.e., commonly used simple diversity indices vs. novel complex measures incorporating ecological information of species (feeding behavior and stream zonation preferences). As a target group, we chose caddisflies, a species-rich, aquatic insect order, in different habitats of an anthropogenically unimpacted, connected karst barrage lake/riverine system. In line with our hypothesis, the complex diversity measures were more efficient in ranking and distinguishing different habitats, particularly the ones with similar communities. We also constructed a novel measure to rank the habitats by sensitivity to climate change, based on diversity of caddisfly communities and vulnerability of species inhabiting them. As expected, the springs were ranked as most vulnerable habitats. Our study further underlines the importance of integrating ecological information into biodiversity and vulnerability assessment of freshwater communities.

**Abstract:**

Freshwater biodiversity is facing a severe crisis due to many human impacts, yet the diversity dynamics of freshwater communities and possibilities of assessing these are vastly unexplored. We aimed at emphasizing different aspects of portraying diversity of a species-rich, aquatic insect group (caddisflies; Trichoptera) across four different habitats in an anthropogenically unimpacted, connected karst barrage lake/riverine system. To define diversity, we used common indices with pre-set sensitivity to species abundance/dominance; i.e., sensitivity parameter (species richness, Shannon, Simpson, Berger-Parker) and diversity profiles based on continuous gradients of this sensitivity parameter: the naïve and non-naïve diversity profiles developed by Leinster and Cobbold. The non-naïve diversity profiles show diversity profiles with regard to the similarity among species in terms of ecological traits and preferences, whereas the naïve diversity profile is called mathematically “naïve” as it assumes absolute dissimilarity between species that is almost never true. The commonly used indices and the naïve diversity profile both ranked the springs as least diverse and tufa barriers as most diverse. The non-naïve diversity profiles based on similarity matrices (using feeding behavior and stream zonation preferences of species), showed even greater differences between these habitats, while ranking stream habitats close together, regardless of their longitudinal position. We constructed the Climate Score index (CSI) in order to assess how diversity and species’ vulnerability project the community’s resistance and/or resilience to climate change. The CSI ranked the springs as most vulnerable, followed by all habitats longitudinally placed below them. We highlight the importance of integrating ecological information into biodiversity and vulnerability assessment of freshwater communities.

## 1. Introduction

Diversity measures have for decades been one of the most important variables for describing and discussing ecological communities or assemblages, and nowadays show even greater merit when examining problems in the age of a rapid extinction of insects [1]. Standard diversity measures such as species richness, Shannon diversity index [2]; Simpson diversity index [3] and Berger-Parker index [4] might each exhibit different projections of diversity from the same given data set. These varying projections are due to the fact that each diversity index considers rare and dominant species in different ways, i.e., each gives different significance to species abundance. A quantitative measure of these differences is given with the sensitivity parameter *q*. This parameter ranges from 0 to ∞, where 0 gives no significance to abundance and is the equivalent to species richness, and where ∞ only considers the abundance of dominant species and is equivalent to the Berger-Parker index. The whole continuous range between these two extremes represents the diversity profile of a community and could be regarded as the fingerprint of the community [5].

One of the main issues with commonly used diversity measures is the presumption of absolute dissimilarity between the analyzed species [5]. In layman terms this means that in a hypothetic freshwater insect community, two different dragonfly species are as similar to one another, as they are to a chironomid species. This approach disregards a whole field of ecological research dedicated to functional-ecological traits, which is especially well-developed for freshwater invertebrates, for instance in the aquatic insect order Trichoptera [6,7,8]. Taking the similarity between species into account gives a more ecologically sound approach and sheds light on unseen relations inherent in the naïve model which presumes absolute dissimilarity between different species [5].

The most important information that comprehensively understood diversity of a certain community has to offer is a measure of community stability, resistance and/or resilience often associated with higher values of local diversity [9,10]. The diversity of aquatic insects changes across geographical and environmental gradients, and still there are many unknowns that need to be addressed, especially considering that the biodiversity of freshwater ecosystems, and therefore aquatic insects, is facing a severe crisis [11]. By fully understanding the different aspects of community diversity, we can help develop insights into the response of aquatic insect communities to the impacts of climate change related to differing diversity dynamics. More specifically, to evaluate if more diverse communities have greater stability and are more likely to endure and/or recover from environmental pressure, such as climate change, more effectively then less diverse communities [10]. However, the knowledge on the impacts of climate change on freshwater aquatic insect communities is largely based on species-level indicators of the potential impacts and vulnerability assessments (e.g., range shifts, phenology changes, etc.; review in [12]), even though these only enable limited prediction of impacts on population and community levels (e.g., [13]).

With over 16,000 species divided into 52 families, caddisflies (Trichoptera) are ranked as the 7th most diverse and species-rich orders of insects worldwide, and the most diverse among primarily aquatic insects [14,15]. Their habitat preferences range from lotic habitats, fast flowing shallow streams and springs to lentic freshwater habitats in their larval phase and surrounding terrestrial habitats in their adult phase [6,16]. Measuring the diversity levels of these insects is usually not only of importance to the knowledge of fundamental ecology, but is also frequently used in determining the ecological status of freshwater habitats, as these insects are considered to be highly valuable indicators of ecological quality in freshwater habitats [17,18,19].

The karst freshwater ecosystems in the Dinaric western Balkan ecoregion (ER5, [20]) have been repeatedly shown to be hotspots for freshwater insect diversity, especially when it comes to the habitat-rich ecosystem complex of barrage lake-forming rivers and streams [21,22,23,24]. As caddisfly communities followed the same trend, exhibiting high biodiversity within these karst ecosystems comprising of springs, different stream types and tufa barriers (e.g., [25,26]), they were chosen as an ideal polygon for the comprehensive testing of different diversity measures.

The main goal of this study was to compare the performance of commonly used indices (that have pre-set values of the sensitivity parameter *q*) and measures of community diversity with non-naïve diversity profiles (that use *q* as a continuous variable) constructed based on different similarity matrices when measuring freshwater diversity. To this end, we first determined how caddisfly community dynamics differ with regard to environmental characteristics of specific karstic freshwater habitat types, and then calculated the commonly used diversity measures for respective communities. Furthermore, we constructed naïve and non-naïve diversity profiles with regard to habitat types. For calculation of non-naïve diversity profiles, we used similarity matrices based on two major life history traits for caddisflies: functional feeding guilds and stream zonation preferences. We hypothesized that these would present more realistic measures of diversity than the traditionally used indices that utilize solely taxonomy-level information. In addition, we aimed at assessing how diversity and specific species’ vulnerability (based on different traits such as endemism, headwater preference, altitude preference, temperature preference, life span) can project the resistance and/or resilience level of the community to pending climate change, by constructing and calculating a new measure of site (ecosystem) vulnerability to climate change or Climate Score index (CSI).

## 2. Materials and Methods

### 2.1. Study Area

**Figure 1:**
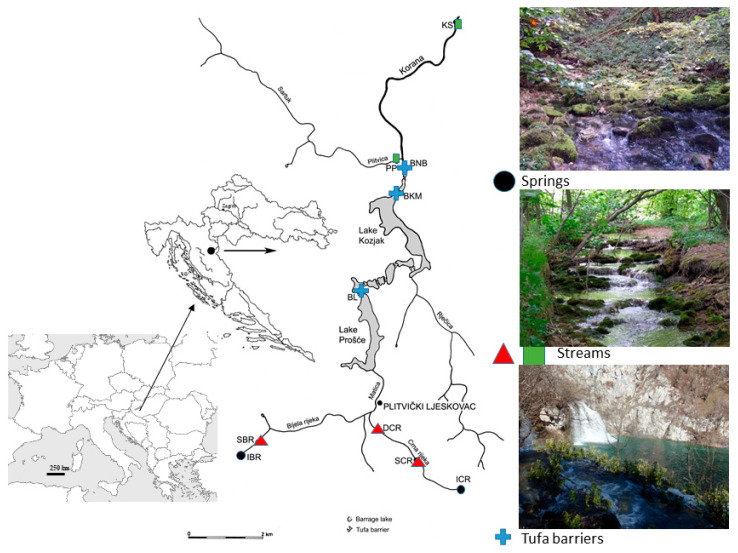
Map of the study area (Plitvice Lakes NP, Croatia) with the locations of the ten sampling sites. Abbreviations of the sampling sites and main site characteristics are presented in Table 1. Springs—**black circle**; Upper stream reaches—**red triangle**; Tufa barriers—**blue cross**; Lower stream reaches—**green rectangle**.

We sampled caddisfly communities at 10 sites in the Plitvice Lakes National Park (Plitvice Lakes NP) in Croatia, located in the karst region of the north-western Dinarides. The Plitvice Lakes NP has a high diversity of different habitat types typical of karst systems, such as springs, streams, lakes and tufa barriers [27]. These habitat types are often characterized by an array of different caddisfly communities [16,25,28,29], and as such provide the perfect background for the comparison of habitat diversity. The Plitvice Lakes are a barrage-lake system, approximately 8.2 km in length, created by numerous tufa barriers. This system is divided into two sections—upper and lower lakes—and is primarily supplied by the Matica stream, formed by the merging of the rivers Crna Rijeka (eng. Black River) and Bijela Rijeka (eng. White River) (Figure 1). According to the Köppen climate classification, this area is influenced by temperate and continental climates. Most of the area is covered by forest consisting of European beech (*Fagus sylvatica* L.) and European silver fir (*Abies alba* Mill.). Our study sites were chosen to encompass four different karstic habitats across a longitudinal gradient: (1) springs, (2) upper streams, (3) tufa barriers and (4) lower streams. General habitat characteristics and main physico-chemical parameters of the water are presented in Table 1.

### 2.2. Field Sampling and Laboratory Analysis

Caddisflies were collected on a monthly basis throughout 2008 (January–December 2008) using six pyramid-type emergence traps at each of the ten sites. Each trap was a 50-cm high, four-sided pyramid with a base of 45 × 45 cm (thus limiting the tested habitat type profile to wadable, lotic parts of the ecosystem such as springs, streams and barriers). Emergence traps were fastened to the streambed, with approximately 2 cm of non-meshed side frame by the streambed that allowed free movement of larvae both in and out of the sampling area. The side frames of each trap were covered with a 1-mm mesh net. On the top of each emergence trap was a collecting container filled with preservative (2% formaldehyde with detergent) [22]. Traps were placed at different substrates present at the sites (listed in Table 1). These substrates and microhabitats were chosen as micro locations representative of the specific sampling site, i.e., with regard to substrate and flow. Six pyramids were placed per site in order to maximize the sampling efforts and compensate for possible specific microhabitat preferences of caddisfly species. Following each sample collection, all material was preserved in 80% ethanol. Taxonomic identification was based on [30] and the systematic review on [31]. Females belonging to the genera *Hydropsyche, Wormaldia* and *Hydroptila* could not be identified to species level with certainty, and are therefore listed as *Hydropsyche* sp., *Wormaldia* sp., and the *Hydroptila occulta* group.

Physico-chemical water parameters (temperature, oxygen concentration, oxygen saturation, conductivity, alkalinity and pH) were measured in situ at each sampling site on a monthly basis in order to portray habitat conditions of caddisfly communities (Table 1). Water temperature, pH and conductivity were measured using hand-held probes (WTW Oxi 330/SET, WTW pH 330 and WTW LF 330, respectively, Hach (Loveland, CO, USA), while alkalinity was measured via titration with 0.1 M HCl with methyl-orange as an indicator.

### 2.3. Data Analysis

In order to evaluate the sampling efforts accomplished for each habitat type in this research, i.e., to estimate the potential undetected species richness, several types of rarefaction curves were calculated (Chao 1, Jackknife 1 and Michaelis-Menten Mean) in PRIMER v7 software package [32]. The overview of estimators and comparison with observed species richness (S obs) per habitat type is given in Supplementary Materials (Figure S1).

#### 2.3.1. Relationship between Caddisfly Communities and Environmental Properties

The relationship between caddisfly communities from different seasonal samples and supporting environmental variables, i.e., physico-chemical water parameters of specific habitat types were tested using canonical correspondence analysis (CCA) in the CANOCO package version 5.0 [33]. The CCA was performed on caddisfly taxa with original abundance data (number of caddisfly individuals per sample). Taxa identified to the genus level were excluded from the analysis if species from the same genus were present at a respective site, otherwise, the *Genus* sp. abundances were included in the analysis. The same was done for all further analyses, in order to portray communities as objectively as possible, with regards to taxonomical bias. Samples with no reported caddisfly individuals (winter months) were also excluded from the analyses. A Monte Carlo test using 999 permutations (*p* < 0.05) was performed to test the significance of the correlations between taxa occurrence (community position) and environmental variables.

#### 2.3.2. Diversity and Similarity of Caddisfly Communities Based on Common Indices

The Shannon, Simpson and Berger-Parker indices, along with species richness, were calculated from caddisfly taxa and abundance data in order to assess the spatial dynamics of caddisfly communities among different habitat types. Plotted mean values and standard deviations of these diversity measures were calculated from all samples of each specific habitat. A similarity percentage (SIMPER) analysis was subsequently performed to determine which caddisfly taxa primarily contributed to the observed similarity within sites. The SIMPER analysis was conducted on the Bray-Curtis similarity matrix. Caddisfly communities were classified in four groups that represent the four major habitat types analyzed in the study (Table 1, Figure 1): springs (sites IBR, ICR); upper streams (sites SBR, SCR, DCR); tufa barriers (sites BL, BKM, BNB) and lower streams (sites PP, KS). This classification was conducted in order to determine diversity and similarity among habitat types with “commonly used” indices and measures. The calculation of biodiversity indices and the SIMPER analysis were conducted using the PRIMER v7 software package [32].

#### 2.3.3. Naïve and Non-Naïve Diversity Profiles Developed by Leinster and Cobbold

For each of the habitat types, a naïve diversity profile was calculated and plotted according to [5], using R software version 3.5.0 [34]. Each diversity profile (curve generated from the diversity measures on a sensitivity parameter gradient) was calculated using the following formula from all samples belonging to a specific site (original monthly samples from each site):

D qZ= (∑​pi(Zp)iq−1)11−q

where: *q* is the sensitivity parameter that controls the relative emphasis placed on common and rare species, and that ranges from 0 to ∞, *^q^D^Z^* is the diversity of order *q* with regard to the similarity matrix (Z) between species, *pi* is the relative abundance (ratio) of species *i* in the community and *Zp* is the relative abundance of species similar to the *i*th—random species (for the naïve model, no species share any similarity between them, and this value equals 0 between every pair of species, except when comparing a species to itself, in which case it equals 1). For details see [5].

The main difference between the naïve and non-naïve diversity profiles is the assumption of absolute dissimilarity between species in the naïve vs. the use of some form of matrices that explain the similarity between species in a specific way in the non-naïve diversity profile. Non-naïve diversity profiles were constructed using different similarity matrices as more realistic measures of diversity. The same formula was followed as in Section 2.3.3; however, with different profiles of caddisfly similarity reflecting some of the most important life history traits of the species. The first similarity profile was based on functional feeding groups (Table S4), while the second was based on stream zonation preferences (Table S5). The stream (longitudinal) preference of invertebrate communities reflects the temperature regime of streams/rivers, and is therefore important for the detection and evaluation of increases in environmental temperature (e.g., due to climate change, [35]). Data on both ecological characteristics was obtained from the freshwaterecology.info database [36]. The similarity matrix among species was calculated using Euclidian distance in the PRIMER v7 software package [32]. Data from feeding preferences and zonation preferences were used as the basis for calculation. Average ratios of functional feeding groups and groups of specific zone preferences are calculated from all samples within a specific habitat type and shown in pie charts next to the non-naïve diversity profiles. The Euclidian distance was considered the most compliant method to use as most species are not strictly “one or the other” in terms of ecological traits. The assignment of taxa to a particular category is based on the ten-point assignment scale as described by [8], in which each species has a small data set, rather than just one number to describe their specific preference. The values of the Euclidian distance were then 1/x transposed and the similarity between two individuals of the same species was set to 1, in order to follow the requirements of the similarity matrix (Z).

#### 2.3.4. Assessing the Resilience of Caddisfly Communities to Climate Change

Depending on the results of the naïve diversity profiles, we used a standard index and combined it with climate change vulnerability scores in order to construct a new index, which would assess how diversity and specific species’ vulnerability can project the resilience of the community and/or its resilience level to future climate change. We argue that habitats with the lowest levels of diversity will be most sensitive to climate change, but also that the specific sensitivity of the species living in the community has to be considered as well (via the climate change vulnerability score). The chosen diversity index (mean value per habitat type) was divided by the sum of relative abundance and vulnerability measures of each species to give a final, newly constructed, measure of vulnerability to climate change or Climate score index (CSI).

The newly constructed Climate Score index (CSI):

CSI=D q1∑​pi*ccvsi

where: *CSI* is the final Climate score index, where lower values indicate higher sensitivity to climate change, *^q^D^1^* is the index of the *q^th^* order chosen after assessing the naïve diversity profile of all habitat types, *pi* is the relative abundance (ratio) of species *i* in the community and *ccvsi* is the climate change vulnerability score of species *i* ranging from 1 (invulnerable) to 7 (highly vulnerable) [ccvsi data from [7] modified from original 0–6 scale of ccvsi specific species value based on different traits: endemism, headwater preference, altitude preference, temperature preference, life span, etc., in order to mathematically match the equation; Table S6]. This and many other freshwater invertebrate environmental trait lists are available for European fauna on https://www.freshwaterecology.info/ accessed on 10 October 2020, [36].

## 3. Results

### 3.1. Relationship between Caddisfly Communities and Environmental Properties

**Figure 2:**
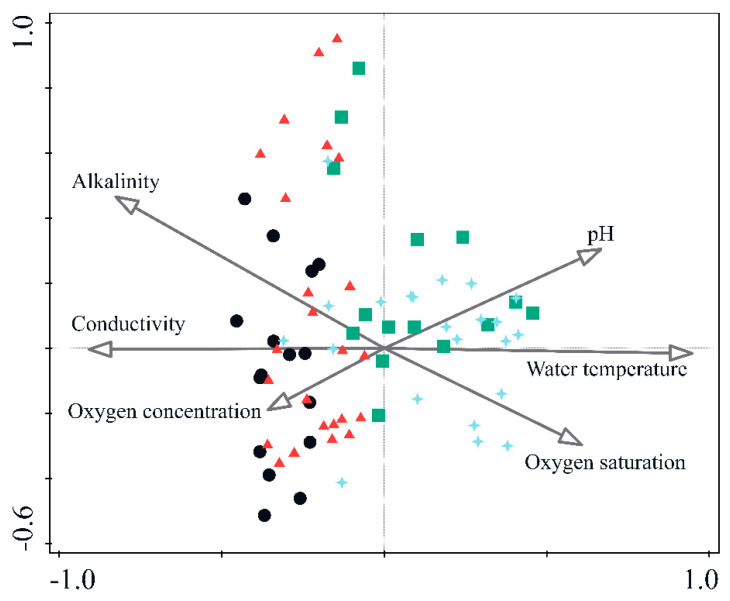
Canonical correspondence analysis (CCA) ordination of caddisfly communities (samples) and physico-chemical water parameters. Physico-chemical water parameters are portrayed with arrows; arrow length indicates the relative importance of the explanatory variables (physico-chemical parameters), and direction—relative to each other and to the sites—indicates positive or negative correlations. Samples (caddisfly communities) from different habitat types are marked as follows: Springs—**black circle**; Upper stream reaches—**red triangle**; Tufa barriers—**blue cross**; Lower stream reaches—**green rectangle**.

A total of 71 caddisfly taxa were recorded from 10 sites belonging to four karst freshwater habitats (see Supplementary Materials; Table S1). In the CCA analysis, the six evaluated environmental parameters explained 21.3% of the total variation of caddisfly communities. The eigenvalues of the first two axes were 0.76 and 0.31 (Figure 2). A Monte-Carlo permutation test showed that the ordination was statistically significant (F = 3.2, *p* = 0.002). Caddisfly communities (monthly samples of individual sampling sites) were clearly grouped in terms of habitat type (some samples even overlapped completely, possibly giving the appearance of a smaller sample number per site). The first axis of ordination indicated an obvious separation between samples (communities) of springs + upper streams against tufa barriers + lower streams. Springs + upper streams were characterized by lower temperature and higher alkalinity, conductivity and oxygen concentration, whereas tufa barriers + lower streams were characterized by higher temperature, pH and oxygen saturation. The main environmental parameter governing the diversity of caddisfly communities was water temperature. A narrow temperature gradient of lower values was observed in springs and upper streams, whereas tufa barriers and lower streams showed a wider temperature gradient of higher values.

### 3.2. Evaluation of Sampling Effort and Species Richness Estimation

Discrepancies in observed species richness compared to estimated species richness are low for springs and upper streams, according to all three estimators of species richness calculated (Chao 1, Jacknife 1 and Michaelis-Menten Mean; Figure S1A,B). Moreover, according to these estimators, between 82% and 95% of species have been collected in these two habitat types within the current study. For lower streams and tufa barriers, however, discrepancies among estimators were higher, predicting that between 66–82% and 52–96% of species richness was sampled in lower streams and tufa barriers, respectively (Figure S1C,D). For overall species richness irrespective of the habitat type, between 84% and 99% of present species were collected based on estimators used (Figure S1E).

### 3.3. Diversity and Similarity of Caddisfly Communities: Common Indices

**Figure 3:**
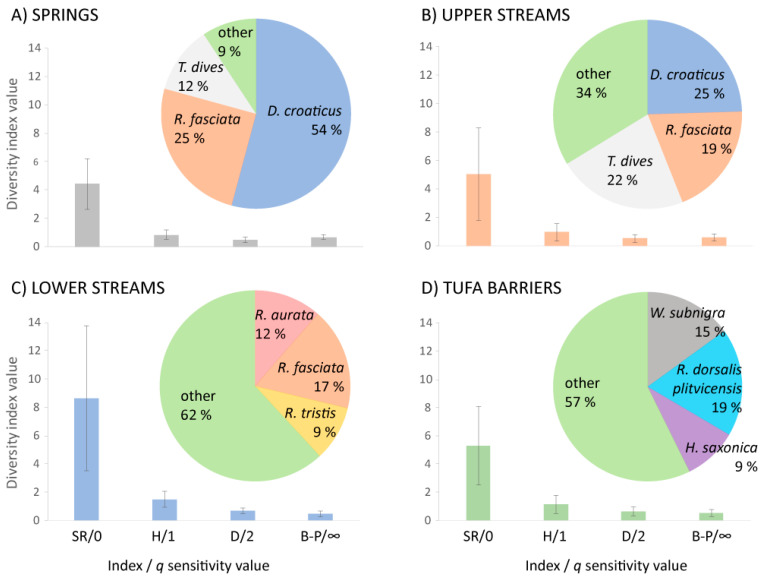
“Classical” (i.e., most commonly used) similarity profiles with values (average ± SD) of familiar diversity indices on a disjunct gradient of the sensitivity parameter *q* shown for each habitat type: (**A**) springs; (**B**) upper streams; (**C**) tufa barriers; (**D**) lower streams. SR = species richness, H = Shannon index, D = Simpson index, B-P = Berger-Parker index. The top three species primarily responsible for the observed similarity within habitat groups (as defined by SIMPER analysis), as well as their ratios of contribution to group similarity are also shown for each habitat type.

The highest average species richness was recorded on tufa barriers (8.65 species), followed by lower streams (5.29), upper streams (5.04) and springs (4.41) (Supplementary Materials; Table S2). All other commonly used indices ranked habitats from highest to lowest diversity as follows: tufa barriers, upper streams, lower streams and springs (Figure 3). The species that contributed most to the within-group similarity (SIMPER analysis; Table S3) were the same for both springs and upper streams: *Drusus croaticus* Marinković-Gospodnetić, *Rhyacophila fasciata* Hagen and *Tinodes dives* (Pictet) (Figure 3A,B). Those contributing most to the similarity observed within the tufa barriers habitat were *Wormaldia subnigra* McLachlan, *R. dorsalis plitvicensis* Malicky and Kučinić and *Hydropsyche saxonica* McLachlan, although this contribution was not as dominant (other species contributed 57%) as in the springs and upper streams (Figure 3C). The taxa that were primarily responsible for the similarity exhibited within the lower stream habitats were all species of *Rhyacophila*: *R. aurata* Brauer, *R. fasciata* Hagen and *R. tristis* Pictet, but again the contribution was not as dominant (other species contributed 62%; Figure 3D).

### 3.4. Diversity and Similarity of Caddisfly Communities: Naïve and Non-Naïve Diversity Profiles

**Figure 4:**
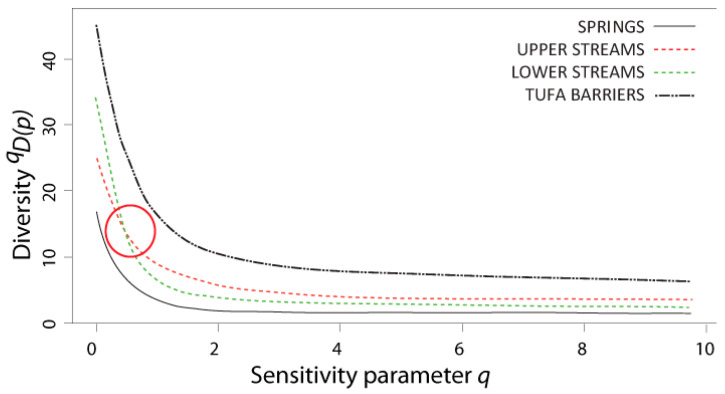
Diversity profiles of caddisfly communities from four different karst freshwater habitat types measured using naïve similarity matrix. The red circle denotes the intersection between the curves for the upper and lower streams.

Naïve diversity profiles were constructed for all four habitat types (Figure 4). The profiles of upper and lower streams intersected somewhere at the sensitivity parameter value of 0 < *q* < 1. Springs exhibited the least diverse community, whereas the tufa barriers showed the most diverse caddisfly community.

**Figure 5:**
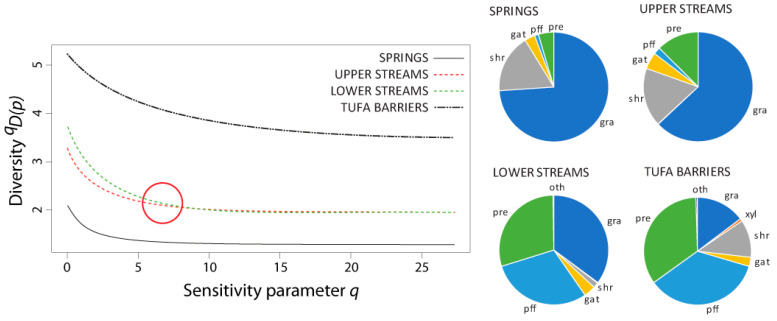
Diversity profiles of caddisfly communities from four different freshwater karst habitat types using a similarity matrix based on functional feeding groups (FFGs). The ratios of specific FFGs in different habitat types are displayed next to the profile. The red circle denotes the intersection between the curves for the upper and lower streams. Abbreviations for feeding types: grazers and scrapers = gra; xylophagous taxa = xyl; shredders = shr; gatherers/collectors = gat; passive filter feeders = pff; predators = pre; other feeding types = oth. (Lower streams: xyl were present with proportion less than 0.2%—not shown here).

The non-naïve diversity profiles for the caddisfly community using the functional feeding group similarity matrix are shown in Figure 5, alongside average ratios of specific functional feeding groups. Springs and upper streams were dominated by grazers and scrapers (75% and 63%, respectively). Grazers and scrapers were also the most abundant functional feeding group in lower streams but, by making up only 35%, were not as dominant as in the first two habitats. Tufa barriers had two, almost equally abundant, feeding groups present: passive filter feeders (36%) and predators (34%). Lower streams were also largely inhabited by passive filter feeders (30%), as well as predators (30%). Gatherers and collectors were present at all habitat types with ratios ranging from 3% to 5%. Xylophagous taxa were present at all habitat types except springs, and their abundances were under 1%. The ratio of shredders exceeded 11% in all habitats except lower streams where they were present in abundances of 2%. The ratio of predators ranged from 5% in springs to 34% at tufa barriers.

**Figure 6:**
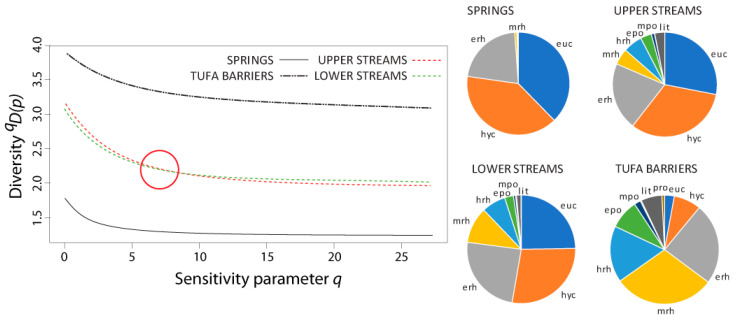
Diversity profiles of caddisfly communities from four different freshwater karst habitat types using a similarity matrix based on specific zone-preferring groups (longitudinal zonation). The ratio of specific species with stream zonation preferences in different habitat types are displayed next to the profile. The red circle denotes the intersection between the curves for the upper and lower streams. Abbreviations for zone-preferring groups (longitudinal zonation): eucrenal = euc; hypocrenal = hyc; epirhithral = erh; metarhithral =mrh; hyporhithral = hrh; epipotamal = epo; metapotamal = mpo; hypopotamal = hpo; littoral = lit; profundal = pro. (Springs: proportion of hyp, epo, mpo and hpo was less than 1%; lower streams: proportion of hpo and pro was less than 1%; tufa barriers proportion of hpo was less than 1%—not shown).

The non-naïve diversity profiles for the caddisfly community using stream zonation preferences as the basis for the similarity matrix are shown in Figure 6, alongside average ratios of specific zone-preferring groups (i.e., average ratios of specific caddisfly species with longitudinal preferences across different habitat types).

In both versions of the non-naïve diversity profiles, the curves for the upper and lower streams intersected somewhere at the sensitivity parameter value of 5 < *q* < 10 (i.e., at *q* ≈ 7.5 and *q* ≈ 6, in the profile based on functional feeding groups and stream zonation preferences, respectively), and both kept the same trendline when increasing the *q* value. As the naïve diversity profile shows, only at the “species richness” level of the sensitivity parameter (*q* = 0) was there a difference in diversity interpretation from the rest of the profile between lower and upper streams.

### 3.5. Resilience of Caddisfly Communities to Climate Change: The Climate Score Index (CSI)

For the calculation of the climate score, we chose the Shannon diversity index (the first index after the intersection of the two profiles, as all other indices that included abundance data in any other form showed the same ranking). The Climate Score Index (CSI) values, i.e., the measure of vulnerability to climate change, showed that springs were most vulnerable to climate change (CSI = 0.187), followed by upper streams (CSI = 0.280), lower streams (CSI = 0.405) and tufa barriers (CSI = 0.734).

## 4. Discussion

Estimates of species richness and diversity largely depend on studied species ecology, appropriateness of sampling method and sampling effort (e.g., [37]), hence, we designed the study in the way to maximize the sampling effort and compensate for possible specific microhabitat preferences of caddisfly species [36]. Estimated species richness in different habitat types encompassed in the current study indicate that the non-detection error was successively minimized by such sampling design, particularly for springs and stream habitats (as up to 95% of estimated species richness was detected). In tufa barriers, however, according to Chao 1 estimator, the efficiency of rare species sampling was the lowest, which is not surprising considering observed community composition and structure in these habitats (see below for detailed discussion [36]. Furthermore, site replication ensures the generality of inference, however, communities always differ between sites even on smallest local scale, particularly when rare species account for large part of species turnover among sites. Thus, absent species will have the same effect as undetected species in diversity estimations, and usually represent an omnipresent limitation in such studies (e.g., [37]).

As presumed, standard diversity measures gave different projections of caddisfly diversity between four habitat types from the same given data set [5,38]. In all cases (using standard diversity measures), the springs in our study had the least diverse community and the tufa barriers the most diverse. Depending on the diversity measure used, upper and lower streams changed in rank, and in general showed similar diversity. When analyzing species within the community, all investigated habitats showed vast differences in community structure; however, with some of the species contributing to relatively high similarity between some of the habitat types. Apart from the species present at the tufa barriers, all species responsible for the high observed similarity within habitat types (based on the SIMPER analysis) inhabit crenal and rhithral stream sections and are lithal habitat specialists [36]. Within the tufa barriers habitat type, the species listed by the latter analysis are rhithral and epipotamal inhabitants and mainly passive filter-feeders [36].

Differences in caddisfly community diversity are thus the reflection of differences in the main functional traits of species composing the respective communities, e.g., stream zonation preferences and feeding behavior [39]. Water temperature was inferred as the main environmental parameter governing the diversity of caddisfly communities (as shown in the CCA), in line with differences in species composition regarding their preference for a particular zone (mainly dependent on the gradient of water temperature, [35,36]). However, the water temperature and the range of its values are a distinguishing property of the specific habitats themselves (and their position in the stream continuum), so there is certainly a strong covariance between the habitat type and temperature in determining caddisfly distribution. This is in concordance with similar research on different macroinvertebrate groups in these habitats [21,24,40].

In the case of diversity profiles constructed solely on taxonomical richness, these show absolute compatibility with the “classical measures” of diversity along with additional information. The profiles of the upper and lower streams intersect, so we cannot definitively conclude which of these two groups of communities harbors higher diversity. If we were most concerned with species richness, we would argue that lower streams exhibited greater diversity [5]; however, the profiles intersected very far to the left (0 < *q* < 1), meaning that from almost any other point of view, diversity was higher in upper streams. The steepness of the curve on the left-hand side of the profiles offers information regarding rare species in the community, i.e., the steeper the drop of the profile, the greater number of rare species in the community, which is here true for all four examined habitats [38]. Finally, the profiles indicate that the caddisfly communities of lower streams are less species-rich but more even in abundance ratios when compared to upper streams. This is in accordance with previous research on caddisfly communities in this barrage lake system [28,39], where community diversity does not follow the common pattern of increase with stream size (e.g., [41]).

In contrast to the naïve diversity profile, both non-naïve diversity profiles (one using a similarity matrix based on functional feeding groups, and the other on stream zonation preferences), similarly placed more emphasis on the diversity differences between tufa barriers and springs, and more or less equalized the diversity value between upper and lower streams. So, if one is principally concerned with dominance, the communities in the lower streams appear to be fractionally more diverse, but from a functional diversity/species richness point of view, there is more diversity in upper streams. However, this could depend on the sampling effort, as the Jacknife1 species richness estimator (based on the species occurring only in a single sample) implies a higher non-detection error for the lower streams than for upper streams, and potentially undetected rare species might have different ecological traits. Moreover, it is important to note here that any reference to diversity values that are similar or equal between communities does not insinuate that these communities do not differ. In comparison to other habitat types, the low values of the non-naïve diversity profiles in springs are the result of numerous crenal specialists that are usually present in these habitats [36,42,43]. The dominance of eucrenal and hypocrenal species and their specific feeding preferences are important as indicators of stable conditions in the crenal habitats, despite decreasing community diversity [43,44]. On the other hand, the most diverse habitats—the tufa barriers—are a unique habitat type featuring a mixture of different habitat types (streams and lake outlets), with high spatial heterogeneity and diversity of food resources [39,45]. As such, tufa barriers can house an array of different lentic and lotic species with a high proportion of filterers and collectors due to specific food sources [39]. As aquatic invertebrate ecology moves more towards analyzing functional- and group-, rather than population-ecology, (mostly as a result of omnipresent biomonitoring efforts) it will become more necessary to develop methods in which we can distinguish rare species from “background noise”, but also have a wide profile of the community diversity fingerprint, i.e., profile.

As all other indices that include abundance data in any other form exhibit the same ranking, for calculation of the climate score index we chose the Shannon diversity index. This is a relatively subjective approach and falls into the category of “depends on the definition of diversity!”. At this point of assessing the final Climate Score Indices (CSI) or vulnerably to climate change between habitats, we encourage researchers to test indices at both sides (*q* values) of the profile crossing over. As ecologists, we cannot ignore abundance in general, but do acknowledge that some assessments of vulnerably to climate change between habitats may be better fitted using indices “left of the profile crossing” (i.e., more focused on species richness than dominance, even species richness itself). This is because many of the commonly used indices are in fact measures of entropy, practically meaning that at higher levels of species richness, communities will appear more similar in terms of the magnitude of the index when compared to lower levels [38]. Our results suggest using the Shannon diversity index when possible (i.e., where the number of species per site is not large and quality abundance data is present), as it considers abundance, while being most sensitive to rare species. Spring caddisfly communities (and species therein) were shown to be most vulnerable to climate change, which is in concordance with other research on caddisflies [46], as well as other invertebrate groups [47,48]. Although different diversity profiles exhibited a high similarity between upper and lower streams, the CSI showed a great distinction between the two, characterizing upper streams as more vulnerable to climate change. Springs and upper stream habitats have a narrow gradient of water temperature oscillation that governs other environmental parameters linked to temperature, such as oxygen saturation [46,49]. Consequently, the proportion of cold-water stenotherm species and species with higher climate change vulnerability potential is higher in these communities. For instance, *D. croaticus*, a dominant species in springs and highly abundant in upper streams, as a micro-endemic and cold-water stenotherm related to crenal sections, fulfills the majority of criteria listed as potentially highly vulnerable to climate change (CCVS score = 4 out of a maximum of 6, [36]). Therefore, such communities are highly vulnerable to climate change, and some authors such as [50] even argue that spring macroinvertebrate abundance may decline by 21% for every 1 °C rise. Moreover, two gradients of climate vulnerability of aquatic insects were inferred for Ephemeroptera, Plecoptera and Trichoptera on the European scale, based on several biological traits; longitudinal gradient (describing successive upstream–downstream features) and a biogeographical gradient (separating endemics from widely distributed taxa) [12].

To conclude, “could we answer a seemingly simple question with a simple answer?”, we show that a simple question turned out not to be so simple at all. However, in the current study, we provide the first evaluation of the performance of commonly used indices and measures of community diversity with non-naïve diversity profiles constructed on similarity matrices incorporating essential ecological knowledge on a freshwater insect group. As such, we provide a novel perspective necessary for more realistic predictions on estimated declines of aquatic diversity due to climate change and anthropogenic alterations (Heino et al., 2009). Our study further highlights the importance of including integrated ecological information when aiming at delivering a relevant biodiversity and vulnerability assessment of freshwater communities [12].

## Figures and Tables

**Table 1:** Characteristics of the sampling sites in the Plitvice Lakes NP. IBR—Spring of Bijela rijeka, SBR—Upper reach of Bijela rijeka, ICR—Spring of Crna rijeka, SCR—Upper reach of Crna rijeka, DCR—Middle reach of Crna rjeka, BL—Tufa barrier Labudovac, BKM—Tufa barrier Kozjak-Milanovac, BNB—Tufa barrier Novakovića Brod, PP—Stream Plitvica, KS—River Korana.

Site		IBR	SBR	SCR	DCR	BL	BKM	BNB	PP	KS
Latitude		N 44°50′05″	N 44°50′04″	N 44°50′10″	N 44°50′22″	N 44°52′17″	N 44°53′39″	N 44°54′07″	N 44°54′07″	N 44°55′33″
Longitude		E 15°33′43″	E 15°33′33″	E 15°36′30″	E 15°35′59″	E 15°35′59″	E 15°36′32″	E 15°36′38″	E 15°36′27″	E 15°37′09″
Stream order		1	1	1	1	2	2	2	1	2
Altitude (m)		720	716	670	667	630	546	504	556	390
Substrate		Pebbles and sand, Macrophytes, Moss	Pebbles and sand, Macrophytes, Moss	Pebbles and sand, Macrophytes, Moss	Pebbles and sand, Macrophytes	Pebbles, Moss on tufa, Tufa with detritus	Pebbles, Moss on tufa, Tufa with detritus, Silt	Pebbles, Moss on tufa, Tufa with detritus, Silt	Pebbles, Moss on tufa, Tufa with detritus, Silt	Pebbles, Moss on tufa, Tufa with detritus, Silt
Water temperature (°C)	min	7.3	7.2	7.1	6.9	2.5	3.1	3.3	3.2	1.7
	max	7.8	9.9	9.7	9.6	20.5	22.9	22.9	15.4	19.8
O_2_ (mg L^−1^)	min	7.6	8.2	7.9	8.8	6.7	8.7	8.4	8.7	9
	max	11.8	11.8	12.5	13.1	12.3	12	12.4	13	14.1
O_2_ (%)	min	65.2	71.2	68.8	96.7	59.7	72	77.3	75.7	79.6
	max	101.8	106.6	115.9	111.1	139.2	113.6	117.1	122.5	121
pH	min	6.9	7.5	7.7	7.9	6.8	6.9	8.2	6.8	6.8
	max	7.8	8.4	8.6	8.4	8.7	8.4	8.7	8.9	8.7
Conductivity (μS cm^−1^)	min	463	472	403	406	366	354	334	409	321
	max	505	498	426	481	426	443	387	444	385
Alkalinity (mg L^−1^ CaCO_3_)	min	235	230	210	215	210	200	185	225	180
	max	295	295	290	280	260	220	230	280	215

## Data Availability

The data presented in this study are available as appendix of this article and on request from the corresponding authors.

## Supplementary Materials

The following are available online at https://www.mdpi.com/2075-4450/12/3/234/s1, Figure S1: Estimates of species richness of caddisflies and sampling efforts in each habitat type in the Plitvice Lakes NP; Table S1: Abundance of caddisfly individuals found at all sampling sites of the Plitvice Lakes (IBR—Spring of Bijela rijeka, SBR—Upper reach of Bijela rijeka, ICR—Spring of Crna rijeka, SCR—Upper reach of Crna rijeka, DCR—Middle reach of Crna rjeka, BL—Tufa barrier Labudovac, BKM—Tufa barrier Kozjak-Milanovac, BNB—Tufa barrier Novakovića Brod, PP—Stream Plitvica, KS—River Korana. Months marked in roman numbering, no individuals at any site present in January; Table S2: Values of “classical” (i.e., most commonly used) similarity indices of caddisfly communities at all sampling sites of the Plitvice Lakes (IBR—Spring of Bijela rijeka, SBR—Upper reach of Bijela rijeka, ICR—Spring of Crna rijeka, SCR—Upper reach of Crna rijeka, DCR—Middle reach of Crna rjeka, BL—Tufa barrier Labudovac, BKM—Tufa barrier Kozjak-Milanovac, BNB—Tufa barrier Novakovića Brod, PP—Stream Plitvica, KS—River Korana. Months marked in roman numbering, no individuals at any site present in January; Table S3: Results of the SIMPER analysis between caddisfly communities at four different habitat types/groups sampling sites of the Plitvice Lakes. Caddisfly communities were classified in four groups that represent the four major habitat types analyzed in the study (Table 1, Figure 1): springs (sites IBR, ICR); upper streams (sites SBR, SCR, DCR); tufa barriers (sites BL, BKM, BNB) and lower streams (sites PP, KS). Only taxa whose contribution to habitat group exceeded 5% are displayed.; Table S4: Taxa specific functional feeding group (FFG) values used in construction of the first similarity matrix. Abbreviations for feeding types: grazers and scrapers = gra; xylophagous taxa = xyl; shredders = shr; gatherers/collectors = gat; passive filter feeders = pff; predators = pre; other feeding types = oth; Table S5: Specific taxa zone-preferring group (longitudinal zonation) values used in construction of the second similarity matrix. Abbreviations for zone-preferring groups (longitudinal zonation): eucrenal = euc; hypocrenal = hyc; epirhithral = erh; metarhithral = mrh; hyporhithral = hrh; epipotamal = epo; metapotamal = mpo; hypopotamal = hpo; littoral = lit; profundal = pro; Table S6: Specific taxa climate change vulnerability scores. ccvsi anging from 1 (invulnerable) to 7 (highly vulnerable) [ccvsi data from [7] modified from original 0–6 scale in order to mathematically match the equation] modified from [36] and used for CSI calculation.
