# Supplementary material for: Peeling the Layers of Caddisfly Diversity on a Longitudinal Gradient in Karst Freshwater Habitats Reveals Community Dynamics and Stability
Source: Insects. 2021 Mar 10;12(3):234. doi: 10.3390/insects12030234 (PMC8001135; doi:10.3390/insects12030234)
Supplement: Supplementary file 1 [file insects-12-00234-s001.zip › insects-1127900-SI/S/Figure .pdf]

(**A**) Springs

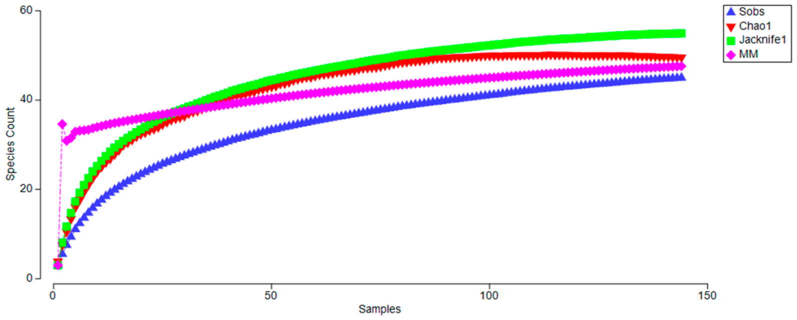

(**B**) Upper streams

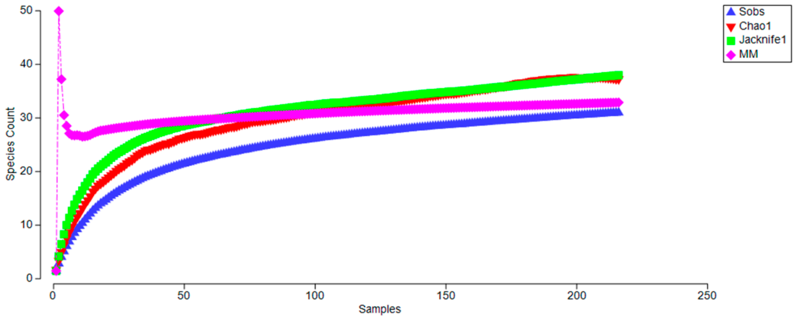

(**C**) Tufa barriers

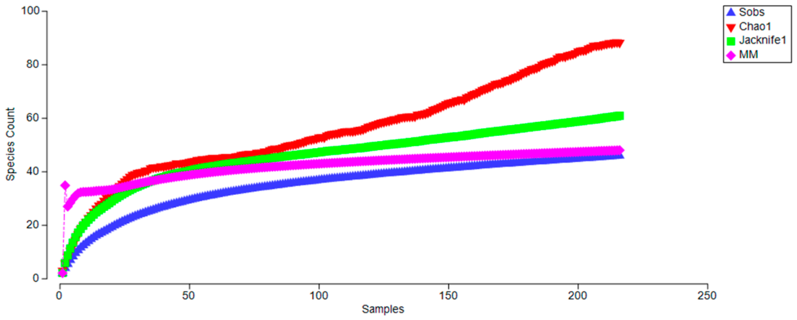

(**D**) Lower streams

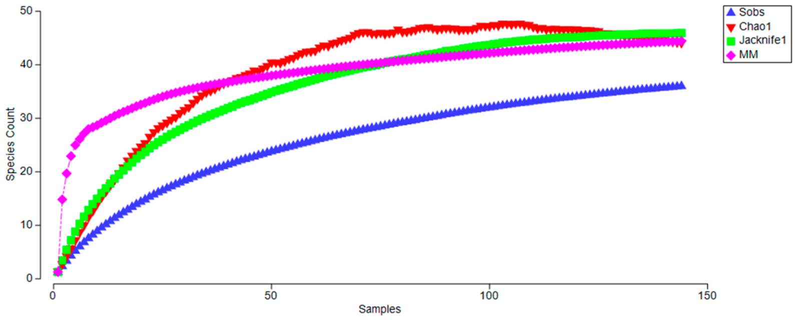

(**E**) All samples

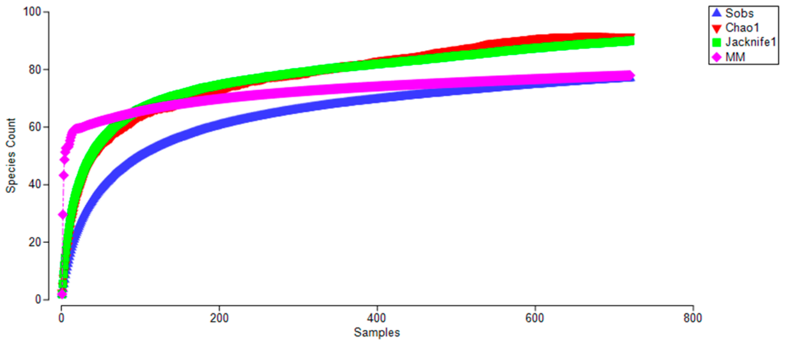

Figure A1. Estimates of species richness of caddisflies and sampling efforts in each habitat type in the Plitvice Lakes NP; (**A**) springs, (**B**) upper streams, (**C**) lower streams, (**D**) tufa barriers, and (**E**) total for all habitat types pooled. (Sobs—number of observed species, Chao1—Chao 1, Jacknife1–Jackknife 1 and MM—Michaelis-Menten Mean).
